# Testicular tumours in children: an approach to diagnosis and management with pathologic correlation

**DOI:** 10.1186/s13244-020-00867-6

**Published:** 2020-05-27

**Authors:** Cinta Sangüesa, Diana Veiga, Margarita Llavador, Agustín Serrano

**Affiliations:** 1 Radiology Department, Pediatric Imaging Section, Hospital Universitario y Politécnico La Fe, Avenida Fernando Abril Martorell 106, 46026 Valencia, Spain; 2 Pathological Department, Hospital Universitario y Politécnico La Fe, Valencia, Spain; 3 Pediatric Urology Department, Hospital Universitario y Politécnico La Fe, Valencia, Spain

**Keywords:** Testicular neoplasm, Ultrasonography, Doppler, Child, Alpha-fetoprotein

## Abstract

Testicular tumours are rare in children. Painless scrotal mass is the most frequent clinical presentation. Tumoural markers (alpha-fetoprotein, beta-human gonadotropin chorionic) and hormone levels (testosterone) contribute to the diagnosis and management of a testicular mass in boys. Ultrasonography is the best imaging modality to study testicular tumours. A benign tumour is suggested when ultrasonography shows a mainly cystic component, well-defined borders, echogenic rim or normal to increased echogenicity lesion when compared to the healthy testicular parenchyma. Malignant tumour is suspected when ultrasonography shows inhomogeneous, hypoechoic, not well-circumscribed or diffuse infiltration lesion. However, these ultrasonographic findings may overlap. Colour Doppler, power Doppler, elastography and contrast-enhanced ultrasonography are useful complementary methods to characterise the focal testicular lesions. Chest computerised tomography and abdominopelvic magnetic resonance are necessary to establish the extension in case of malignant proved tumours.

Benign tumours are more frequent in prepuberal boys and malignant tumours in pubertal boys. Mature teratoma prepubertal-type is the most common histologic type. Testicular sparing surgery is the choice in benign tumours. Radical inguinal orchiectomy is indicated in malignant tumours. Prognostic is excellent.

The purpose of our study is to show an approach to the diagnosis and management of the most frequent testicular tumours in children according to clinical manifestations, imaging findings and tumour markers levels based on histologically confirmed tumours in our hospital.

## Key points

- Testicular tumours are rare in children.
- Benign tumours are more frequent in prepuberal boys and malignant tumours in pubertal boys. Teratoma is the most common histologic type.
- Ultrasonography is the best imaging modality to diagnose testicular tumours. MR can be necessary as an adjunct method when scrotal US findings are uncertain.
- Tumoural markers are necessary to study a testicular mass.
- Testicular sparing surgery is the choice in benign tumours.

## Introduction

Testicular tumours (TTs) are rare in children under 15, then accounting for 2–4% of all childhood cancers [1, 2]. They have two peaks of incidence in paediatric population: neonatal and puberty. This rise of incidence over the age of 9 could be due to the high hormone levels at puberty. The malignant potential of germinal cell tumours (GCTs) increases rapidly after this age, while benign tumours are more frequent in younger boys [3].

**Fig. 1:**
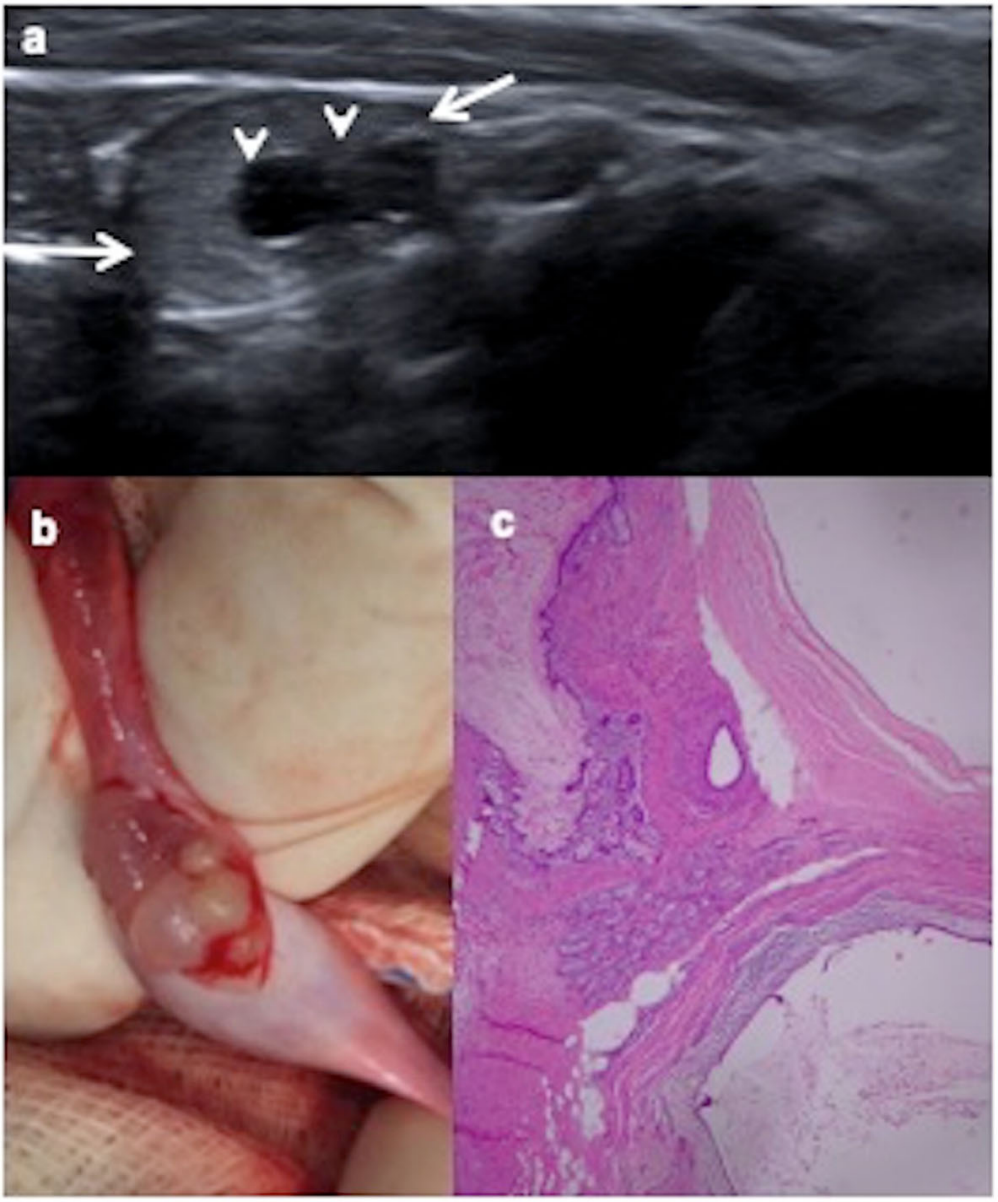
Cystic mature teratoma in cryptorchidism. A 10-month-old boy with right cryptorchidism. **a** Sonography identifies the right testis (arrows) in the inguinal canal presenting two cystic lesions (arrowheads). **b** Surgical procedure shows the testis during tumourectomy where cystic lesions are visible. **c** Cystic mature teratoma is the definitive histopathologic result. Microscopic view of the tumour shows squamous, digestive and ciliated columnar epithelium. No immature elements are identified

Intrautero exposures, perinatal variables and risk factors have been evaluated. An increased risk of TT is associated with cryptorchidism and gonadal dysgenesis [3–6]. Mature teratoma is the most frequent tumour (83%) in paediatric intraabdominal testes (Fig. 1). Risk factors for development of malignancy in cryptorchidism are bilateral, abnormal external genitalia and late or uncorrected undescended testis; seminoma is the most frequent malignant tumour associated to cryptorchidism [7]. Gonadal dysgenesis present in the disorders of sexual differentiation has a high risk to develop TT (35–50%), thus requiring prophylactic gonadectomy. Gonadoblastoma occurs exclusively in gonadal dysgenesis and seminoma is the most frequent malignant tumour in it [8].

**Fig. 2:**
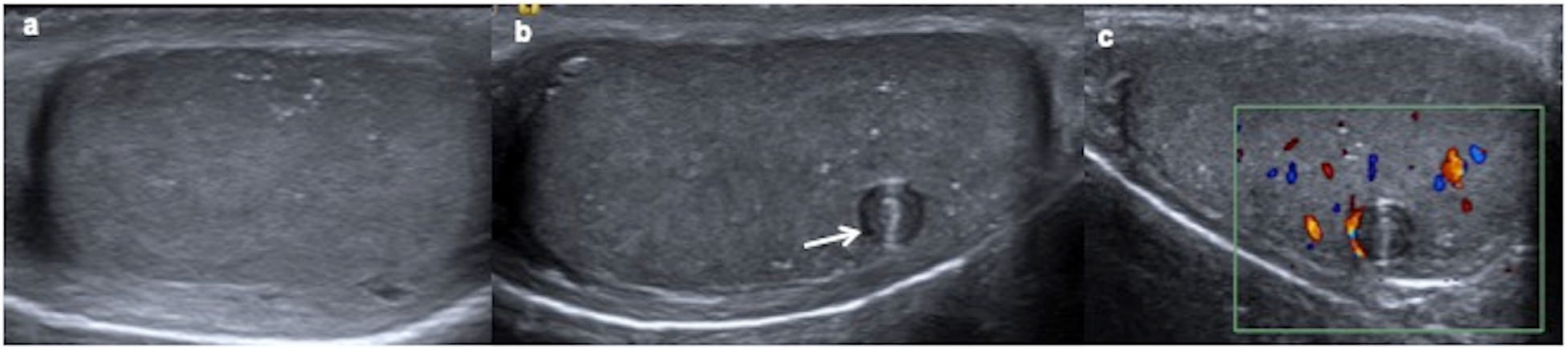
Tumour and microlithiasis. A 12-year-old boy with right scrotal pain. **a** Longitudinal ultrasound view of the left testicle shows multiple punctuate non-shadowing echogenicities compatible with microlithiasis. **b** Longitudinal view of the right testicle shows besides microlithiasis, a round well-defined lesion with onion ring appearance (arrow) typically characteristic of epidermoid cyst. **c** Colour Doppler shows only peripheral vascularization. An intraoperative biopsy diagnoses an epidermoid cyst and a testis sparing surgery is practised

Testicular microlithiasis (TM) defined by five or more non-shadowing intratesticular echogenic calcific foci have shown association with testicular tumours in children (Fig. 2) [9], although the contribution of TM to the risk of malignancy is controversial and there is not any agreement for the management and monitoring of children with TM [10, 11]. Most preadolescent boys with TM and testicular tumour have other predisposing conditions for testicular cancer as cryptorchidism [9].

It has been proposed that tumourigenesis depends on the permissive action of gonadotropins in the testis, as gonadotropins levels follow the same pattern of age-adjusted incidence of testicular cancer. The disease would start in foetal life, with alterations in primordial cells formation, giving rise to neoplasia in situ cells that remain quiescent until they are stimulated by gonadotropin-mediated signals [12]. According to the World Health Organization (WHO), TTs are divided into seven groups (Table 1). A pathogenetic differentiation between two types of yolk sack tumours and teratomas is carried out: postpubertal, which are derived from a precursor lesion (germ cell tumours derived from germ cell neoplasia in situ—GCNIS), and prepubertal, (unrelated or non-GCNIS) [13].

**Table 1:** The 2016 WHO classification of testicular tumours

Germ cell tumours unrelated to germ cell neoplasia in situ (non-GCNIS)	Spermatocytic tumour Yolk sac tumour, prepubertal type Teratoma, prepubertal type - Dermoid cyst - Epidermoid cyst - Differentiated neuroendocrine tumours Mixed teratoma and yolk sac tumour, prepubertal type
Germ cell tumours derived from germ cell neoplasia in situ (GCNIS)	Seminoma Embryonal carcinoma Yolk sac tumour, postpuberal type Choriocarcinoma Teratoma, postpuberal type Mixed germ cell tumours Regressed germ cell tumours
Sex cord-stromal tumours	Leydig cell tumour Sertoli cell tumour Granulosa cell tumour
Tumour containing both germ cell and sex cord-stromal elements	Gonadoblastoma
Haematolymphoid tumours	
Tumours of collecting duct and rete testis	
Miscellaneous	

Prepubertal-type teratomas (50%) and prepubertal yolk sac tumours (15%) are the most frequent TTs in children. Others tumours are epidermoid cyst (15%) and stromal tumour (Leydig cell and Sertoli cell), which account for approximately 10% [14, 15]. Some series describe yolk sac tumour as the most frequent because benign tumours are not included [2]. However, our personal experience is that prepubertal-type teratomas are the most frequent TT during childhood following the yolk sac tumour. Mixed germ cell tumours rarely occur in prepuberal boys while in postpuberal boys most tumours are malignant and mixed germ cell tumours are present in greater numbers [15, 16].

Ultrasonography (US) is the imaging modality of choice for studying TTs with a 100 % sensitive and a negative-predictive value of almost 100% [11, 17]. Magnetic resonance (MR) is used as a supplemental imaging technique in exceptional cases where scrotal US findings are inconclusive or non-diagnostic, in the evaluation of abdominal cryptorchidism and in the extension of a histologically confirmed malignant TT [18, 19].

An approach to the TT diagnosis is based on ultrasonographic findings, clinical and endocrinological data and tumour marker levels as alpha-phetoproteine (AFP), beta-human gonadotropin chorionic (B-HCG), lactate dehydrogenase (LDH) or testosterone [2, 17].

The purpose of our study is to show an approach to the diagnosis and management of the most frequent TTs in children according to clinical manifestations, imaging findings and tumour markers levels based on histologically confirmed tumours in our hospital.

## Clinical presentations

TTs usually manifest as painless testicular mass (82–90%) and less than 10% as painful mass secondary to haemorrhage or necrosis [2]. Physical exploration and other clinical data (fever, acute pain, vomiting) can help to differentiate an intratesticular lesion from hydrocele, inguinal hernia, testicular torsion or inflammatory scrotum.

TTs can be detected on a prenatal US; the most frequent congenital TT is juvenile granulosa cell tumour, the left testis is the most affected and up to 20% have ambiguous external genitalia [2, 20–22].

Some histologic types can show more specific clinical presentations: Leydig tumour has a peak incidence between 5 and 10 years and the dysregulation of hypothalamic-pituitary-testicular axis may lead to hormonal stimulation because of the androgen and oestrogen secretion by the tumour, and near 30% of cases present signs of feminizing or virilizing syndrome (pubertal precocious, gynecomastia). Testosterone high levels are present [19, 23, 24]. Bilateral testicular Leydig cell tumours are rare (3%) and require exclusion of Peutz-Jeghers syndrome. Sertoli tumour in children can produce gynecomastia typically. The subtype large-cell calcifying Sertoli tumour is seen in prepuberal boys associated with syndrome Peutz-Jeghers and Carney complex being then frequently multifocal and bilateral [2, 14, 19, 25].

Incidental TT smaller than 2 cm occurs in about 13% of children and goes up to 30% when children are between 5 and 12 years old [14, 16].

The most common secondary tumour is the leukemic infiltration. The typical clinical presentation is as unilateral or bilateral painless testicular mass. The scrotum size is larger than in any other primary TT [26].

## Imaging findings

US is the first imaging technique to study testicular masses, with a sensitivity of almost 100% but with low specificity because the differentiation between benign and malignant neoplasms is difficult in most cases [2, 10, 17]. TTs are predominantly homogeneous hypoechoic, but can also be heterogeneous with solid, cystic or calcific components that reflect the underlying histologic characteristics [18].

Doppler and Power colour demonstrate the elevated blood perfusion in most malignant masses while benign tumours are normally well-circumscribed with decreased blood flow [2, 22]. Then, Doppler ultrasound findings may mimic focal or diffuse orchitis and clinical data are necessary for the diagnosis [27].

EFSUMB (European Federation of Societies for Ultrasound in Medicine and Biology) guidelines recommend the contrast-enhanced ultrasound sonography (CEUS) for the discrimination of focal testicular lesions. CEUS can display microvascularization, and the hyperenhancement is an indicator of malignant tumour. CEUS can be useful to confirm the absence of vascularity in benign complex and epidermoid cysts [28].

Elastography may increase diagnostic accuracy by assessing the stiffness of the lesions. Malignant neoplastic lesions are harder because the density of tumoural cells and vessels is greater than in normal testicular tissue [29, 30].

MR can be necessary in rare cases where scrotal US findings are inconclusive or non-diagnostic. Malignant germ cell tumours spread first via lymphatic, usually through the inguinal ring and spermatic cord to the retroperitoneum; thus, the lymphatic pathway should be included in the initial study with abdominopelvic MR [31, 32]. Choriocarcinoma has an early haematogenous spread; chest CT is necessary [18].

Testicular cancer has staging systems based on pathology after orchiectomy or tumourectomy, radiology with chest CT and abdominopelvic MR, and serum tumour markers [31] (Table 2).

**Table 2:** Staging system of testicular and paratesticular malignant tumours (Children’s Cancer Group and Paediatric Oncology Group)

Stage I	- Tumour limited to testis with negative microscopic margins, completely resected by high inguinal orchiectomy - Normalised tumour markers - Tumour capsule cannot have been violated by needle biopsy, incisional biopsy or tumour rupture - No clinical, radiographic or histologic evidence of disease beyond the testes.
Stage II	- Complete orchiectomy with violation of the tumour capsule - Microscopic disease in scrotum or high in spermatic cord (< 5 cm from proximal end) - Failure of tumour markers to normalise or decrease with an appropriate half-life
Stage III	Retroperitoneal lymph node involvement > 2 cm
Stage IV	Distant metastases

We show the imaging features with emphasis on the ultrasonographic findings of the most frequent TTs in children following WHO classification of 2016.

### Germ cell tumours unrelated to germ cell neoplasia in situ (non-GCNIS)

Most TTs in children belong to this group, i.e. tumours that are not derived from intratubular cell germ. They are named as prepuberal-type tumour.

### Yolk sac tumour, prepubertal type

Yolk sac tumour, also known as endodermal sinus tumour, is the most frequent testicular malignant tumour in children under 2 years [2, 33]. Prepuberal yolk sac tumour is normally pure form [13]. Elevated AFP is present in 95–98% of the cases, with it being used as a tumoural marker for its diagnosis and follow-up to check for regression or recurrence of the tumour [2, 14, 33].

**Fig. 3:**
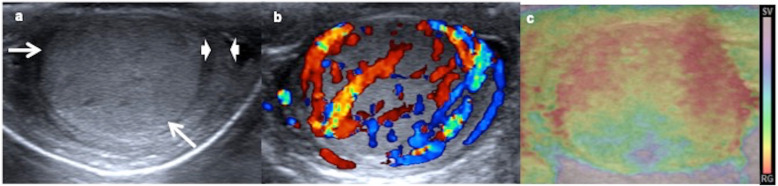
Yolk sac tumour. A 15-month-old boy with a painless swollen left testicle. **a** Longitudinal view of the left testis shows near-complete replacement by a hypoechoic solid mass (arrows) with a rim of normal parenchyma (arrowheads). **b** Power Doppler reveals increased blood flow within the tumour. **c** Elastography shows heterogeneous with high strain of the mass. The boy has a very high level of alphaphetoprotein and inguinal orchiectomy is practised. Yolk sac tumour is the definitive pathologic diagnosis

US findings show a large round or ovoid focal or diffuse solid hypoechoic homogeneous mass that can entirely occupy the affected testis, and sometimes the only US finding may be testicular diffuse enlargement [18, 32, 33]. Rarely heterogeneous with necrosis and calcifications areas are described [2, 32, 33]. They are typically hypervascular tumours on colour Doppler and predominantly stiff lesions on ultrasound elastography (Fig. 3) [29].

**Fig. 4:**
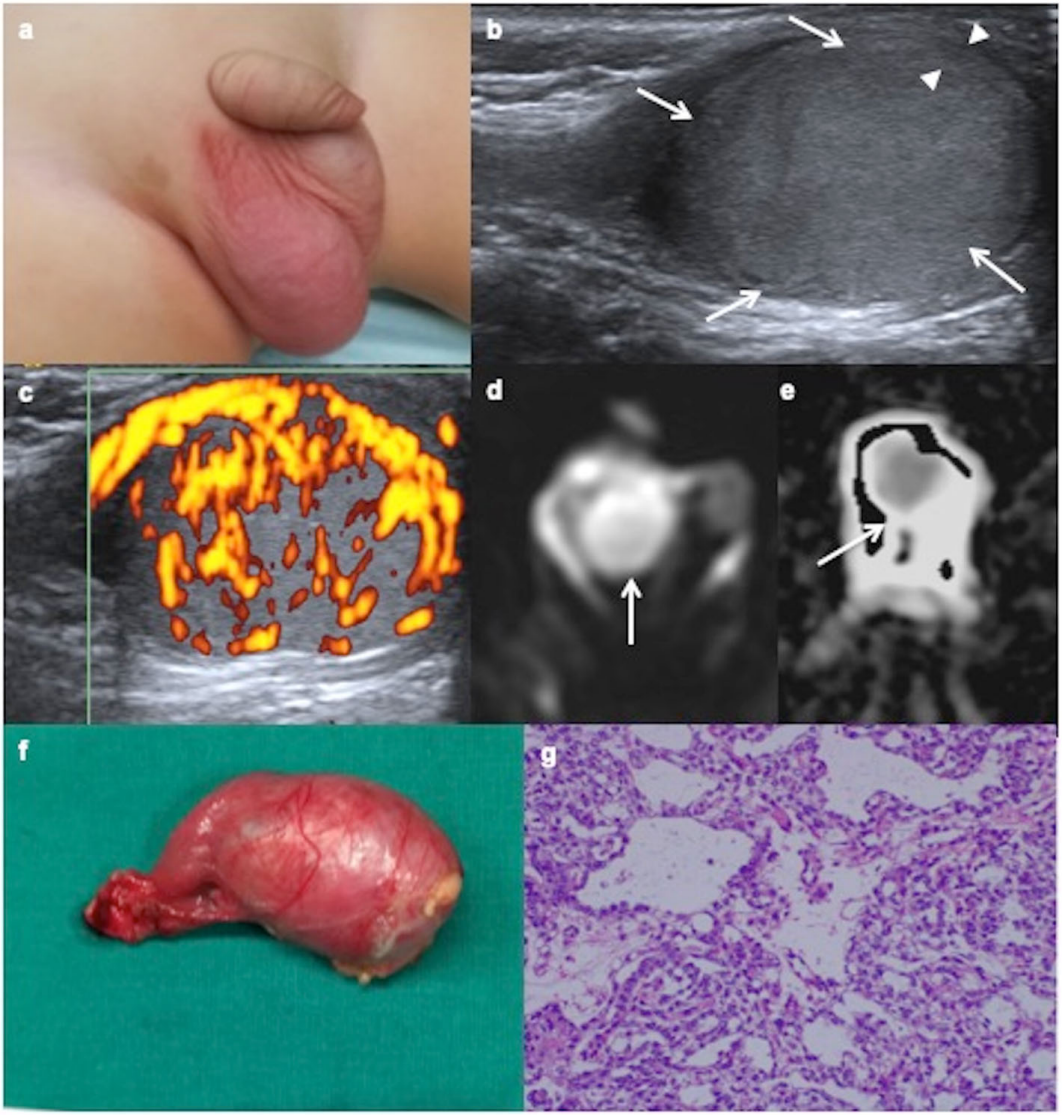
Yolk sac tumour. **a** A 14-month-old boy with a painless right scrotal mass. **b** Ultrasound shows an intratesticular hypoechoic mass (arrows) with a rim of healthy tissue preserved (arrowheads). **c** Power Doppler reveals abnormally abundant blood flow within the tumour. **d** Axial diffusion-weighted MRI shows the mass with hyperintensity (arrow). **e** ADC map shows a low signal intensity from diffusion restriction of the mass (arrow). The boy has a very high level of alphaphetoprotein and inguinal orchiectomy is practised. **f** Surgical piece includes inguinal cord. **g** Microscopic exam shows a microcystic yolk sac tumour containing glands and tubular structures with subnuclear vacuoles.

**Fig. 5:**
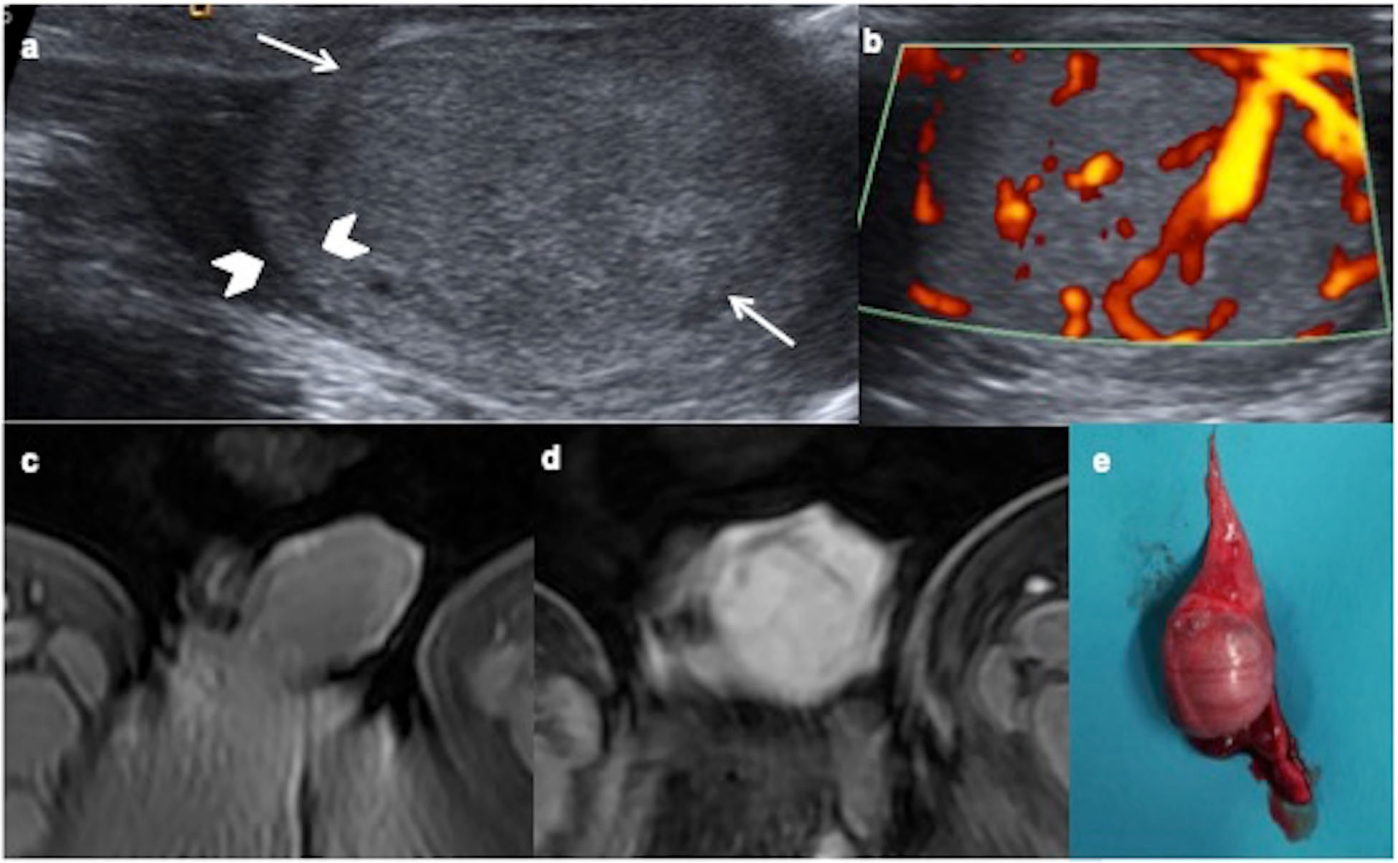
Yolk sac tumour. A 19-month-old boy with a painless swollen right testicle. High level of alphaphetoprotein (3.450 ng/mL). **a** Longitudinal view of the right testis shows near-complete replacement by a hypoechoic solid mass (arrows) with a rim of normal parenchyma (arrowheads). **b** Power Doppler reveals increased blood flow within the tumour. **c**, **d** Axial fat-supressed T1 show a hypointense mass with avid enhancement of the tumour after the contrast. **e** Surgical piece after the radical inguinal orchiectomy

MR findings are poorly described. Our experience in the cases practised in our hospital show hypointensity in T1, hyperintense in T2, enhancement after contrast and restriction diffusion with low ADC (apparent diffusion coefficient) (Figs. 4 and 5).

### Teratoma, prepubertal type

It is the most frequent TT in children. Teratomas are complex tumours derived from all three germ layers (endoderm, mesoderm and ectoderm). Prepubertal-type teratomas are those non-associated with GCNIS; they do not have significant cellular atypia and no metastasis [13]. They are divided into mature (contain exclusively adult cells) and immature (contain embryonic or foetal cells) [13, 14].

**Fig. 6:**
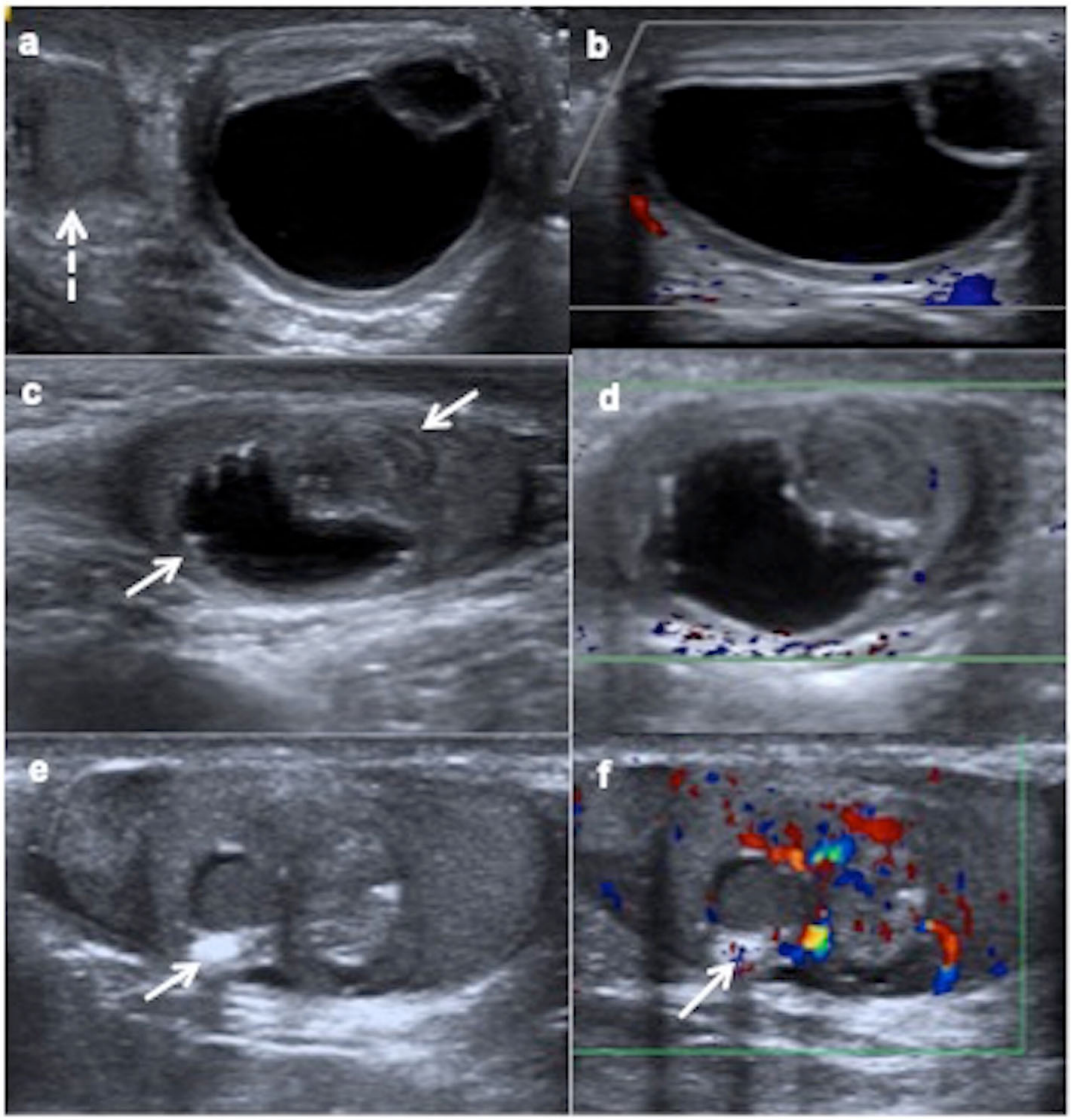
Mature teratomas, prepubertal-type: ultrasound appearances. **a**, **b** Ultrasound and colour Doppler of a 10-month-old boy with a swollen and tender left testis show an avascular cystic mass completely replacing the testicular parenchyma, an orchiectomy being required. Right testis (discontinuous arrow). **c**, **d** Ultrasound and colour Doppler of a 8-month-old boy with a painless scrotal left mass show a mixed solid-cystic mass (arrows) without blood flow in the solid part of the lesion. Orchiectomy is practised because of the big size of the lesion. **e**, **f** Ultrasound and colour Doppler of a 8-year-old boy with a swollen and hard right testis show a heterogeneous solid mass, calcifications (arrow) and blood flow inside. A testis sparing surgery is practised since sufficient salvage parenchyma is visible

**Fig. 7:**
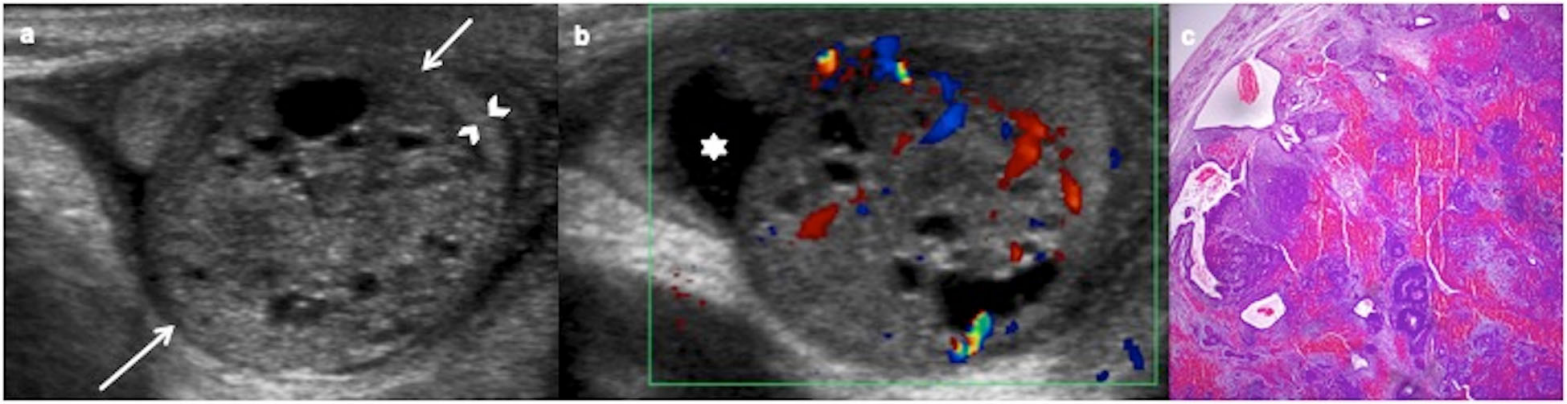
Immature teratoma, prepubertal-type. A 31-day-old boy with a painless left scrotal mass. **a** Ultrasound shows a heterogeneous predominantly solid mass (arrows) replacing the testis except a peripheral rim (arrowheads). **b** Colour Doppler demonstrates vascularization in the solid components of the mass. Hydrocele (star). Radical inguinal orchiectomy is practised. **c** Microscopic exam shows stromal, neural, cartilage and digestive immature cells

On US, they generally form heterogeneous masses, well defined, single- or multiseptated, with complex architecture depending on the components of the three germinal layers. They may show cystic parts, which can have different echogenicity depending on the content (mucoid, keratinous serous), but also peripheral solid parts of cartilage, fibrosis, scars or calcifications (Fig. 6). The imaging features of mature and immature teratomas are usually overlapped, although mature teratomas are predominantly cystic with 93% sebaceous fat content, whereas immature teratomas are larger, encapsulated, with solid areas composed of neuroectodermal components (Fig. 7). Teratomas are poorly or mildly vascularised on colour Doppler. Inhomogeneous elastogram map is due to the heterogenous cellular structures [14, 15, 18, 29].

CT and MR are rarely practised. The demonstration of fat, calcification, cystic component inside the lesion approaches the diagnosis.

Dermoid and epidermoid cysts are included in the category of prepubertal-type teratomas [13].

### Dermoid cyst

Dermoid cyst is composed of pilosebaceous units; it may have adjacent lipogranulomas but no other germ cell layer. On US, a cyst lesion with a thin wall is seen but the final diagnosis is pathologic [13].

### Epidermoid cyst

**Fig. 8:**
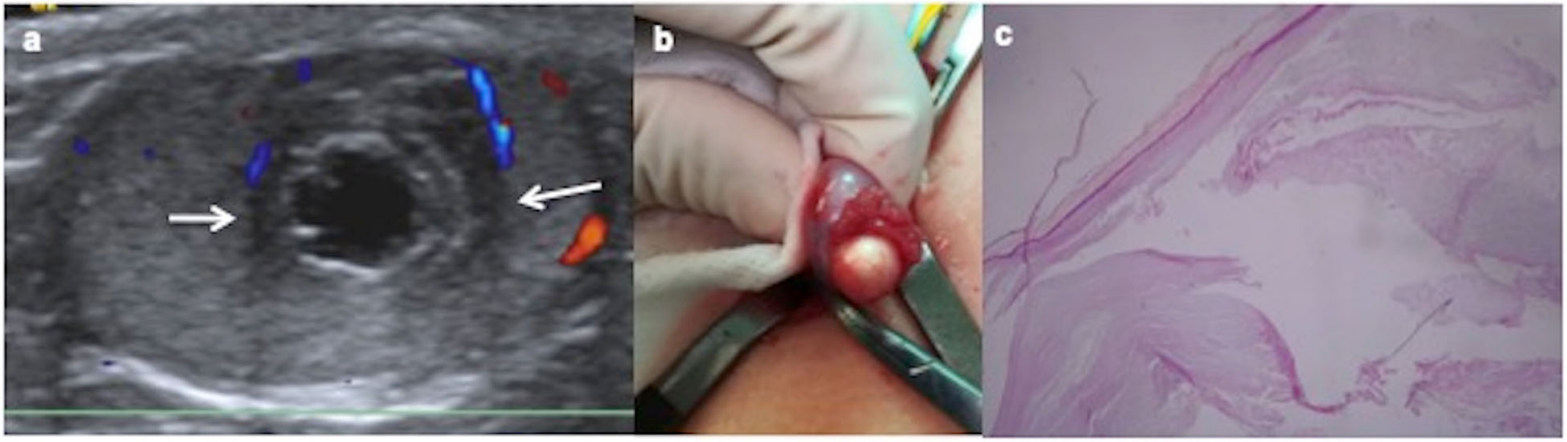
Epidermoid cyst. A 9-year-old boy with scrotal traumatism several days ago. **a** Longitudinal view of ultrasound colour Doppler image of the left testis shows an avascular intratesticular mass with alternating hyperechoic and hypoechoic rings (onion ring) (arrows). **b** Enucleated lesion seems a pearl because of its keratin component. A testis sparing surgery is practised. **c** Microscopic view of the pathologic specimen shows the cyst lined by squamous epithelium

Epidermoid cyst is composed of benign squamous epithelium producing keratin with lamellar target appearance but no other germ cell layers. Pathological findings correlate with radiological features [26]. On US, the typical appearance is a well-circumscribed avascular heterogeneous mass with concentric rings of hypoechogenicity and hyperechogenicity (“onion ring” appearance) due to the keratin layers and cyst component inside (Fig. 8). Less frequent US shows a target appearance as a hypoechoic mass with a hyperechoic rim, sometimes calcified, depending on the compactness, the maturation and the disposition of the keratin [2, 18, 34–36]. Atypically, the cyst can contain cheesy material and may mimic a solid tumour [32]. Epidermoid cyst demonstrates hard elastographic properties because of its composition [29].

On MR, epidermoid cyst can be on T1 hypointense due to keratin debris or hyperintense due to lipid content, and hyperintense on T2 because of the content of lipids and water. The outer fibrous capsule is hypointense on T1 and T2 sequences. No enhancement is seen after contrast [2, 18, 35].

### Germ cell tumours derived from germ cell neoplasia in situ (GCNIS)

**Fig. 9:**
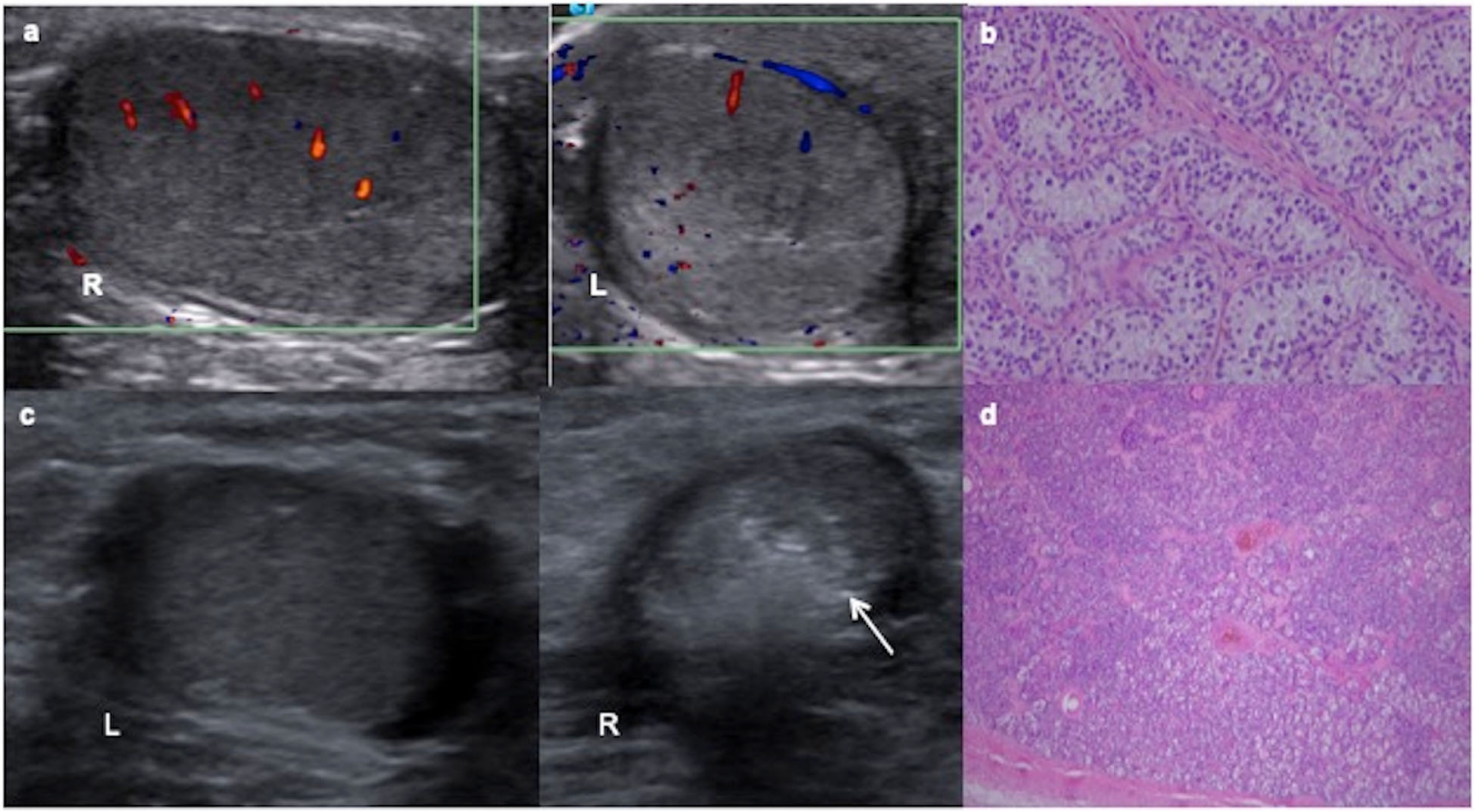
Two cases of intratubular germ cell neoplasia. **a** A 12-year-old boy with left cryptorchidism (inguinal testis). Colour Doppler ultrasound shows asymmetric size with normal right (R) testis and left (L) testis smaller, heterogenous and predominantly hyperechoic. **b** The surgical inguinal approach checks a hard cryptorchidic testis. Orchiectomy is practised and microscopic view of the pathologic specimen shows a large atypical cells with clear cytoplasm angulated nuclei with coarse chromatin, prominent nucleoli and cell borders resembling “fried egg” seminoma cells. **c** An 11-year-old boy with right cryptorchidism (inguinal testis). Ultrasound shows a normal left testis (L) and a small and heterogenous hyperechoic right testis (R) with small calcifications (arrow). **d** It is extirped giving rise to an intratubular germ cell neoplasia or carcinoma in situ of the testis: Spermatogenesis is absent and dystrophic calcifications are seen inside seminiferous tubules

GCNIS or intratubular germ cell neoplasia is considered the precursor of most adult malignant germ cell tumours. Very rare in children, GCNIS or intratubular germ cell neoplasia can be found in cryptorchidism testes (5–8%) and testes extirped by gonadal dysgenesis in childhood. This is considered a premalignant lesion to remain dormant until puberty, and later on these abnormal cells develop either along a unipotential or the totipotential gonadal cell line. No characteristic imaging findings are described because they are pathologic findings without testicular enlargement, but in our own experience, the cryptorchidic testis is always smaller and with heterogenous echogenicity predominantly hyperechoic (Fig. 9) [18].

#### Seminoma

Testicular seminoma originates in the germinal epithelium of the seminiferous tubules. It may contain syncytiotrophoblastic cells, thus producing a low elevation in serum B-HCG [13]. Seminoma is uncommon in children, and the malignant neoplasm most related to cryptorchidism [27].

**Fig. 10.**
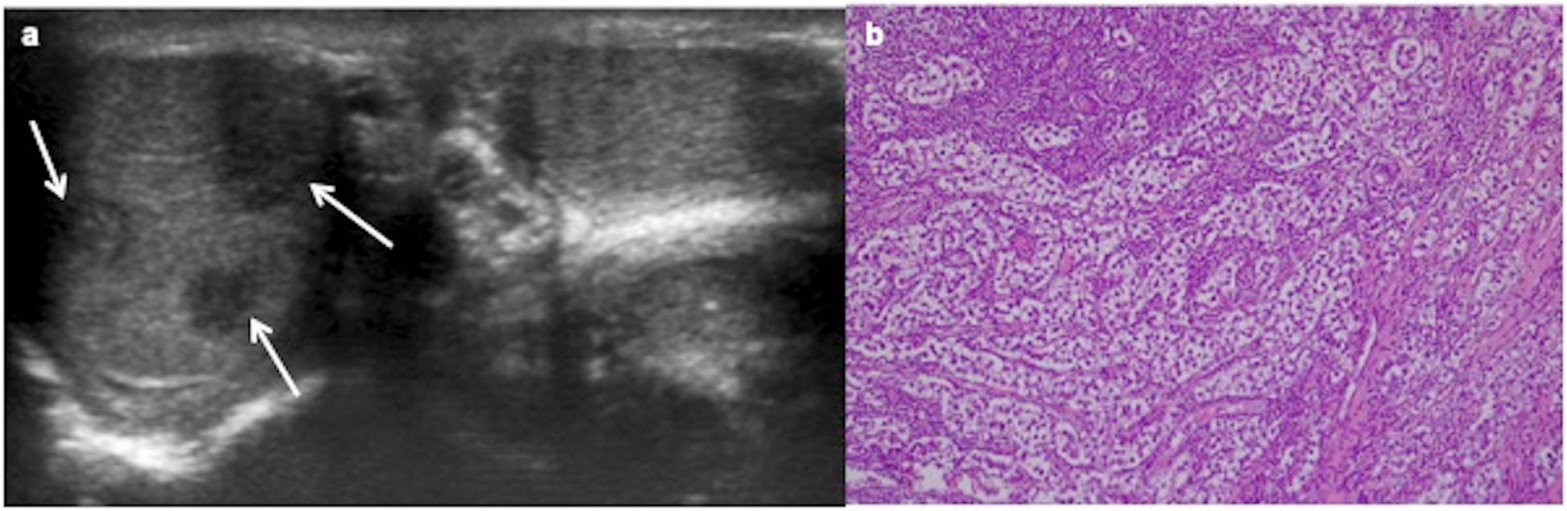
Seminoma. A 13-year-old boy with a right testicular mass since two months ago. **a** Transverse ultrasound scan of both testes shows an enlarged right testis with multinodular hypoechoic mass (arrows). Intraoperative biopsy diagnoses seminoma and an inguinal orchiectomy is practised. **b** Sheets of relatively tumour cells within fibrous bands and lymphocytic infiltrate

On US, seminoma is typically round and homogenously hypoechoic and can be lobulated or multinodular (Fig. 10) [18], although it can be heterogeneous when it grows. Neither calcification nor cystic components are present and they seldom break the tunica albuginea [26, 27]. Doppler shows mainly peripheral vascularization. On strain elastograms, seminoma displays a uniformly stiff nature [29].

On MR, seminoma is multinodular homogeneous hypointense on T2 with fibrovascular septa that enhance contrast and restriction diffusion with low ADC map [19].

#### Embryonal carcinoma

**Fig. 11:**
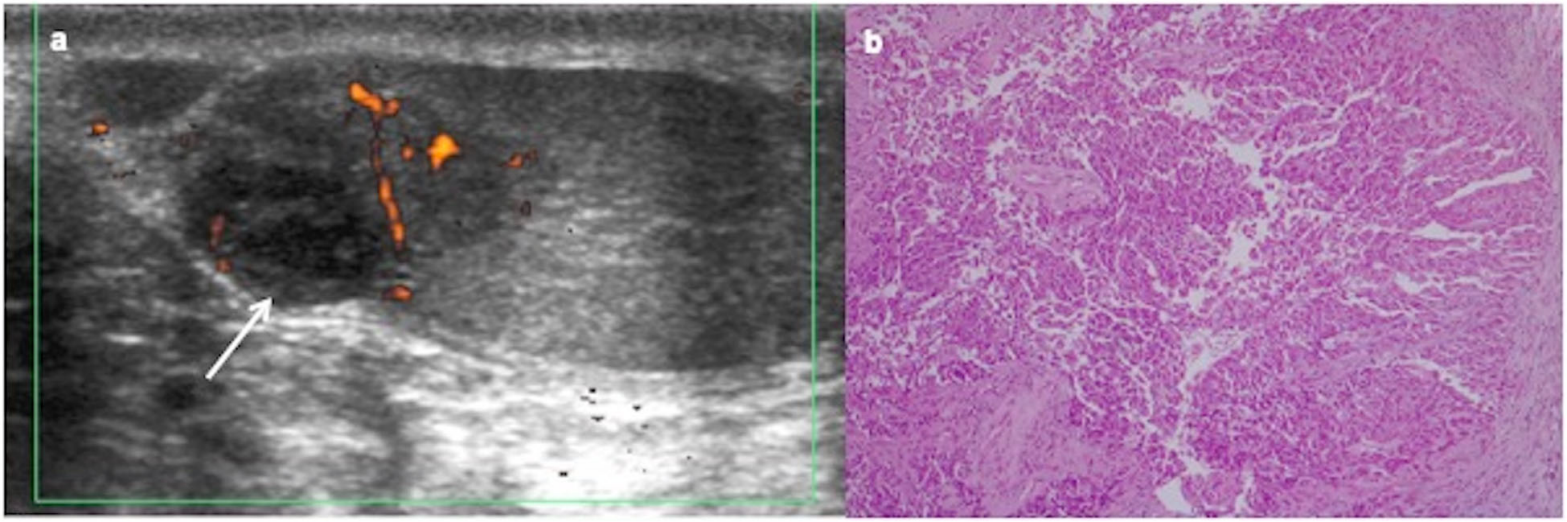
Embryonal carcinoma. A 12-year-old boy with right testicular mass since 2 weeks ago. **a** Longitudinal ultrasound view of the right testis and colour Doppler show hypoechoic mass with growth invading the tunica albuginea (arrow) and a very slight increased perfusion. A radical inguinal orchiectomy is practised after intraoperative biopsy. **b** Atypical polymorphic cells with glandular pattern and infiltration tunica albuginea

It arises from primitive anaplastic tumour cells. Although normally smaller in size, embryonal carcinoma is more aggressive and may invade the tunica albuginea (Fig. 11). On US, it is a poorly circumscribed heterogeneous lesion [18, 26]. On MR imaging, embryonal carcinoma is heterogeneous with necrosis and poorly marginated [19].

#### Choriocarcinoma

Choriocarcinoma is a malignant trophoblastic tumour composed of cytotrophoblastic and syncytiotrophoblastic cells producing high serum levels of B-HCG, thus resulting in a good tumoural marker to diagnosis [26]. On US, it is a predominantly hypoechoic lesion with ill-defined margins [17]. It can metastasise to distant sites, such as lung, liver, brain or gastrointestinal tract; it is the most aggressive histological subtype. Metastases are normally haemorrhagic and present contrast-enhanced CT or MR [18, 31].

#### Mixed germ cell tumours

Non-seminomatous germ cell tumours may be pure or mixed cell type [13]. Mixed cell tumours are more frequent in puberal boys. On US, they appear as heterogeneous ill-defined lesions that may present internal cysts, necrosis or calcifications [26]. These tumours show inhomogeneous elastogram map due to their heterogenous cellular structures and necrosis [29].

#### Regressed germ cell tumours

It is very rare in children and also known as “burnt-out” germ cell tumours where a rapidly growing testicular tumour can outgrow its blood supply and atrophy. Most frequent histological types of GCNIS with this behaviour are seminoma and choriocarcinoma [37, 38]. Pathological findings include a scar, GCNIS in the adjacent parenchyma and intratubular coarse calcifications [13]. On US, they are small and can be hypo or hyperechoic, or they may be seen as a focal calcification. Extensive retroperitoneal lymphadenopathies are frequent in the moment of the diagnosis and they may represent seminomatous or non-seminomatous extragonadal germ cell tumours [18].

### Sex cord-stromal tumours

Stromal tumour accounts for approximately 10% of testicular neoplasm in paediatric population. They include Sertoli, Leydig and juvenile granulose cell tumours [22, 27].

#### Leydig cell tumour

Leydig cell tumours are the most common type of sex cord-stromal tumour and arise from interstitial (Leydig) cells.

**Fig. 12:**
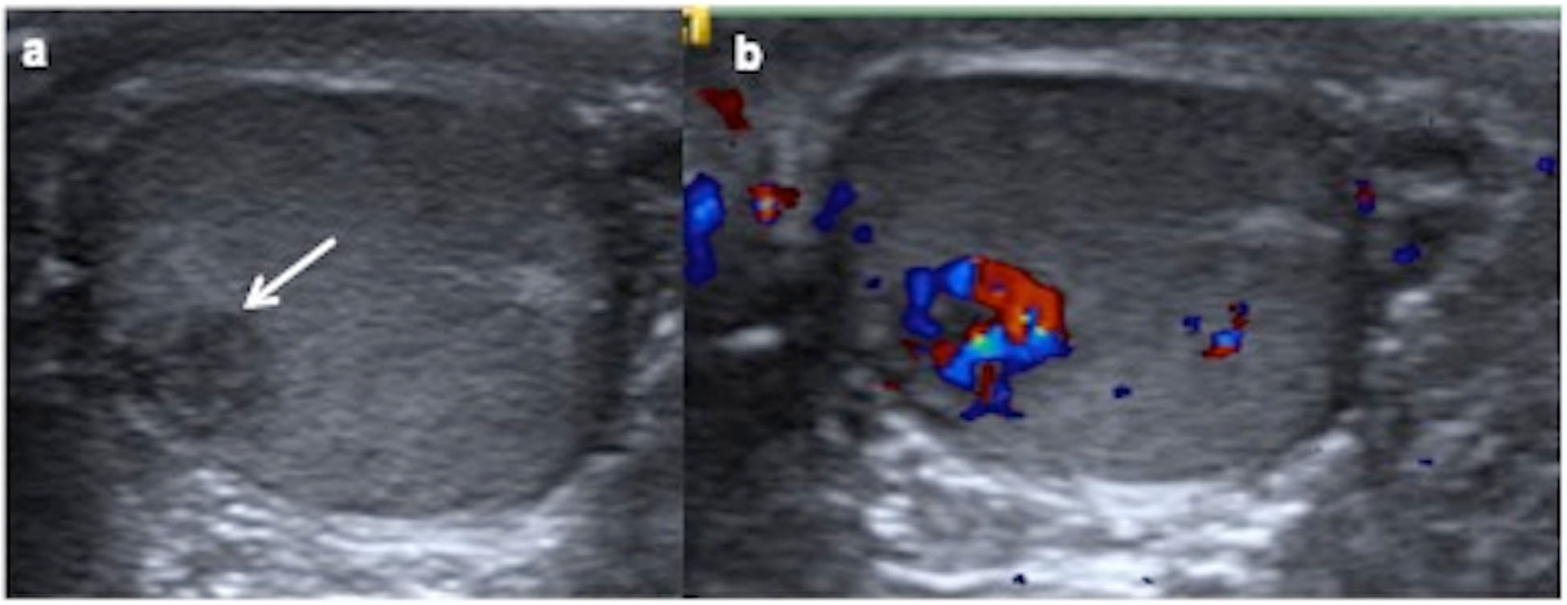
Leydig cell tumour. A 7-year-old boy with precious puberty. High level of testosterone (3.762 ng/mL). **a** Transverse ultrasound view of the right testis shows a hypoechoic lesion (arrow). **b** Colour Doppler imaging demonstrates an increased tumour vascularization. A testis sparing surgery with tumour enucleation is practised. Leydig cell tumour is the definitive pathologic diagnosis

**Fig. 13:**
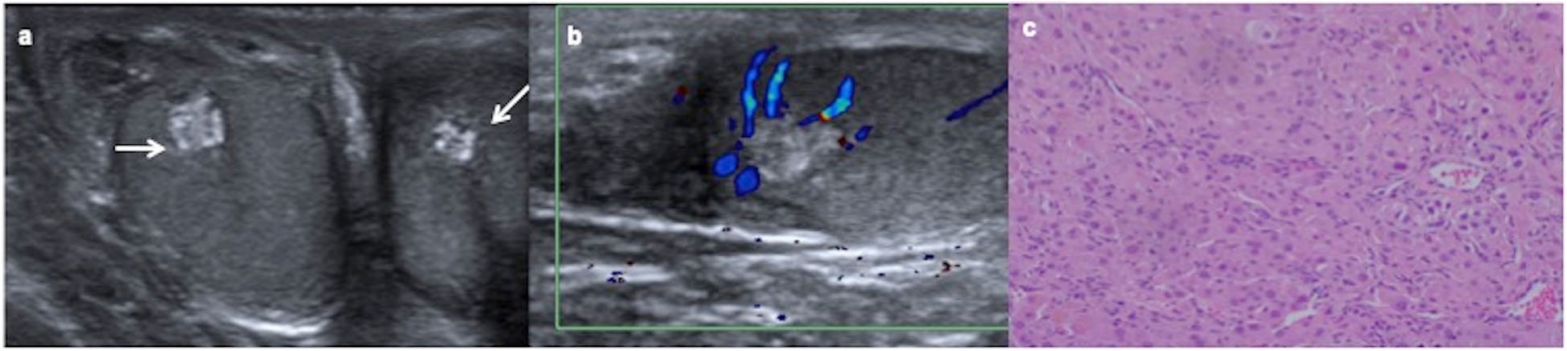
Bilateral Leydig cell tumour. A 9-year-old boy with Peutz-Jeghers syndrome. **a** Transverse ultrasound view of both testes shows intratesticular hyperechoic lesions (arrows). **b** Longitudinal colour Doppler view of the right testis demonstrates no blood flow in the lesion. Bilateral intraoperative biopsy is practised resulting in Leydig cell tumour in both testes. Bilateral tumourectomy is carried out. **c** Microscopic view of biopsy in both testes show the same pattern: diffuse large polygonal cells with abundant eosinophilic cytoplasm, round nuclei and prominent nucleoli corresponding to Leydig tumour

**Fig. 14:**
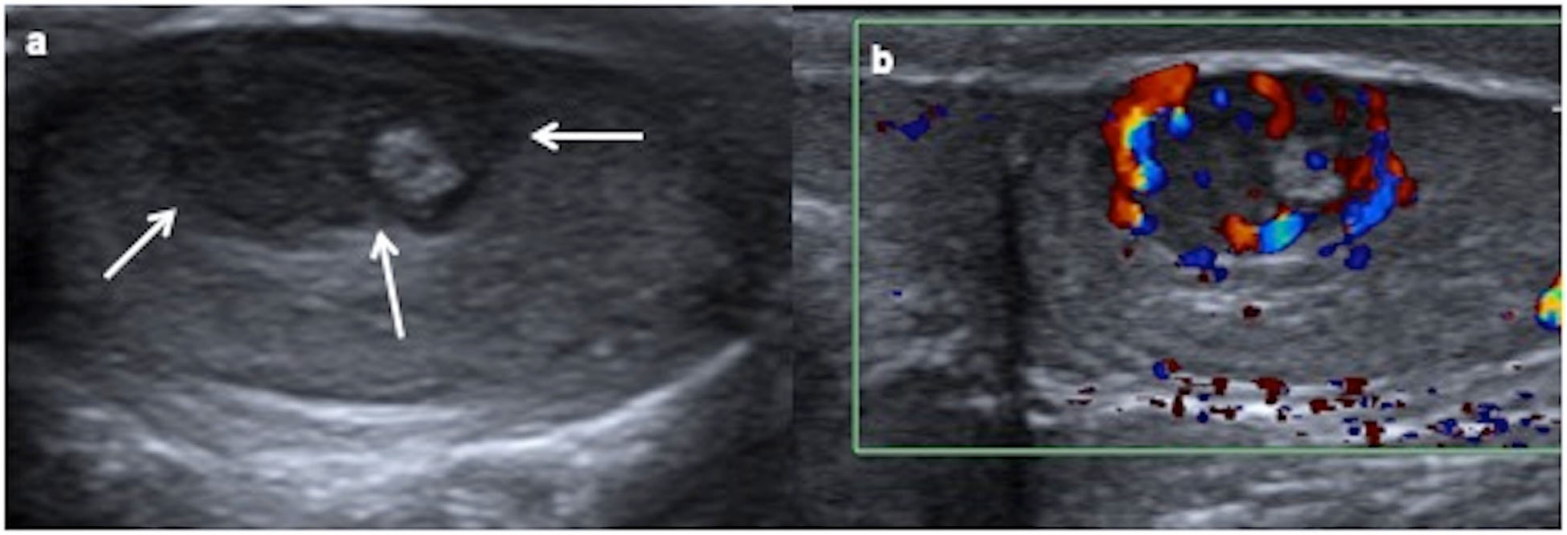
Leydig cell tumour. A 7-year-old boy presents precocious puberty with a high level of testosterone. Scrotal ultrasound is performed. **a** Longitudinal view of the left testis shows a well-delimited lobulated heterogeneous mass (arrows) and (**b**) colour Doppler image shows peripheral and central vascularization of the mass. Intraoperative biopsy diagnoses a Leydig cell tumour and a testis sparing surgery is carried out

On US, it is an isolated hypoechoic small solid mass, sharply defined, and normally located in the periphery of the testicle (Fig. 12) [23, 27, 32, 39, 40]. It is less frequent as hyperechoic lesion in case of bilateral Leydig tumour (Fig. 13) [19, 40], while large lesion can be lobulated with mixed echogenicity (Fig. 14) [39].

Colour Doppler demonstrates a hypervascularised lesion [39]. In case of a small tumour, CEUS allows to demonstrate arterial hypervascularization compared to the surrounding parenchyma with a rapid fill of contrast bubbles distinguishing from focal scars or atrophic areas. Leydig tumour shows anelastic stiffer qualities but varies from mildly hard to hard on strain elastography, as described in the literature [29, 40].

On MR, Leydig tumour is usually isointense on T1-weighted images and hypointense on T2-weighted images and mild contrast enhancement [19].

#### Sertoli cell tumour

Sertoli cell tumour originates within the seminiferous tubules Sertoli cells. There are three types: classical, large-cell calcifying Sertoli cell and sclerosing Sertoli cell. Classical Sertoli cell tumour is a round well-defined echogenic mass. The large-cell calcifying Sertoli cell tumour subtype is the most frequent one in children, it being hyperechogenic, partially calcified, multiple and bilateral, usually associated with Carney syndrome and Peutz-Jeghers syndrome. Benign tumours are small and usually seen in younger patients, while malignant tumours are commonly larger than 4 cm, necrotic or haemorrhagic and can metastasise to retroperitoneal lymph nodes [18, 19, 25]. Sertoli cell tumours can present mildly hard to hard on strain elastography [29].

On MR, Sertoli tumour usually demonstrates homogeneous intermediate signal on T1, hyperintense signal on T2, and homogeneous enhancement [19].

#### Juvenile granulosa cell tumour

Juvenile granulosa tumour cell is the most frequent congenital testicular tumour. It shows reticular appearance with follicle-like structures filled with mucoid material.

**Fig. 15:**
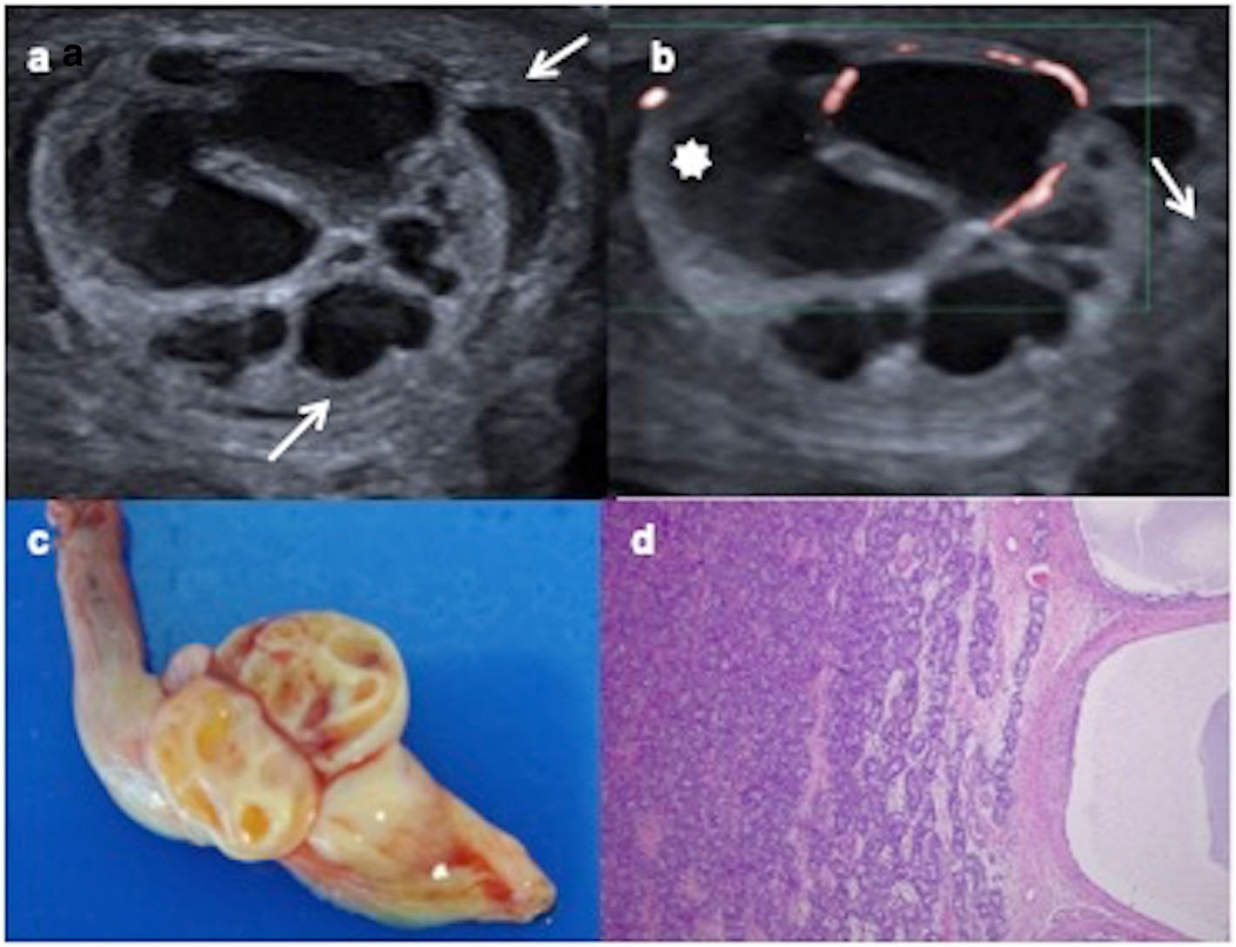
Juvenile granulosa cell tumour. A 16-day-old male with a swollen scrotal left mass since birth. **a** Testicular ultrasonography demonstrates a multiseptated cystic intratesticular mass (arrows) replacing the testicular parenchyma. Hydrocele (star). **b** Colour Doppler shows vascularization in the thickened septations. **c** Surgical piece after radical inguinal orchiectomy showing tumour and spermatic cord. **d** Microscopic appearance: many layers of granulosa cells with oval nuclei and abundant cytoplasm in a multilocular mass

On US, it presents a characteristic aspect as a well-circumscribed non-invasive multilocular cystic mass with thick septations, or less frequently as a solid mass with intralesional cysts. Colour Doppler US shows hypervascularity of the solid components and septations (Fig. 15) [21, 22].

On MR, juvenile granulose cell tumour is hypointense on T1 images, hyperintense on T2 sequences, and with enhancement of wall and septations after contrast administration [19, 21].

### Tumour containing both germ cell and sex cord-stromal elements

#### Gonadoblastoma

Very rare and more frequent in undescended testes or gonadal dysgenesis. Imaging findings are not well-defined. On US, they are described as multiple hyperechoic lesions [41].

### Secondary tumour

#### Leukemic infiltration

**Fig. 16:**
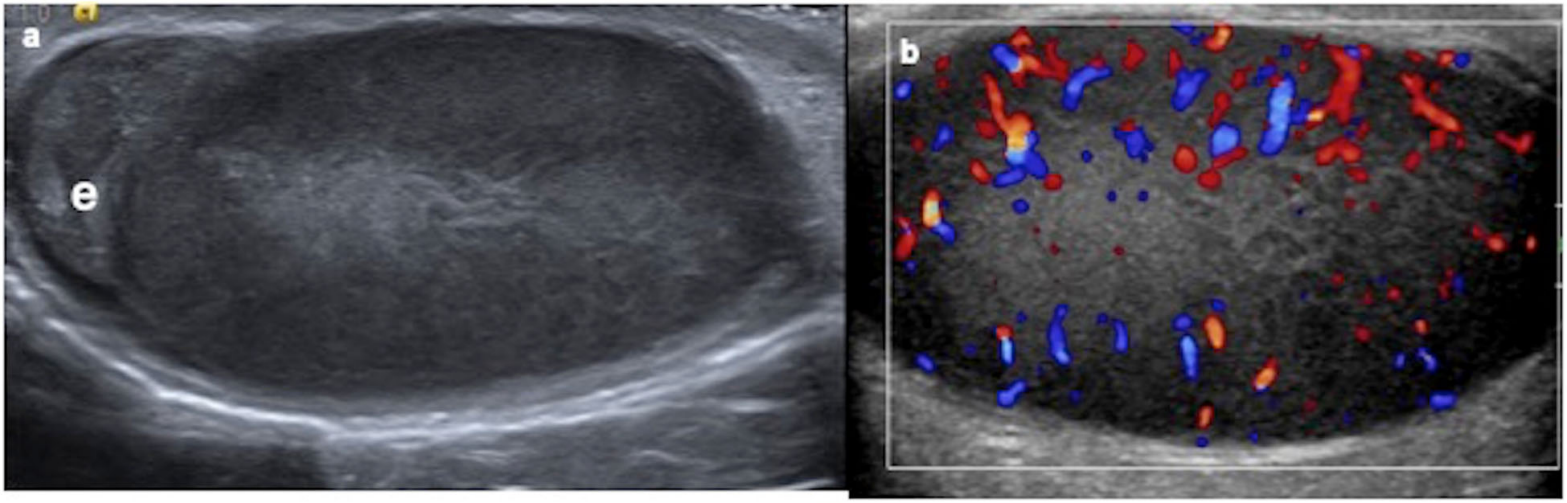
Testicular leukemic infiltration. A 8-year-old boy diagnosed of leukaemia with a right swollen testis. **a** Longitudinal ultrasound view shows an enlarged and diffuse hypoechoic right testis. e (epididymis). **b** Colour Doppler demonstrates increased vascularization

Unilateral or bilateral, testes present a big size, them being extremely hypoechoic in ultrasound with important hypervascularity on colour Doppler (Fig. 16) [33].

## Management

After being clinically suspected and once the ultrasonographic exam confirms an intratesticular lesion, the determination of serum markers (AFP, B-HCG), hormonal levels (testosterone) and LDH are necessary to guide the diagnosis and the treatment. It should be taken into account that infants under 12 months can show high AFP levels physiologically [2, 10, 14]. Percutaneous testicular biopsy is not usually performed because of the risk of lymphatic seeding [42].

Testicular sparing surgery should be used in children with a TT in which the normal testicular tissue seems salvageable on US and with normal tumoural markers. Intraoperative frozen section examination can be applied to confirm pathological tumour as well as to justify conservative surgery [14, 16].

Since prepubertal-type teratomas are benign, testicular sparing enucleation is recommended in prepuberal boys. Epidermoid cysts can be treated with tumourectomy when they present a classic ultrasonographic imaging or the lesion is smaller than 3 cm [26]. Leydig cell tumour is always benign in children and treatment must be as conservative as possible. In exceptional cases where malignant Leydig tumour is diagnosed (10%), orchiectomy is necessary [10, 23, 24, 39, 43]. Juvenile granulosa tumour is considered a borderline tumour, and although radical inguinal orchiectomy is normally practised, a tumour enucleation could be sufficient when healthy testis tissue is present [22].

When specific tumoural markers are high (AFP in case of a yolk sac tumour, BHCG in case of a choriocarcinoma) inguinal radical orchiectomy is planned. An incision is made in the groin and the urologist removes the entire tumour along with the testicle and the spermatic cord. The spermatic cord contains vas deferens, vascular vessels and lymphatic vessels that can act as pathway for spreading to the rest of the body. Adjuvant chemotherapy in malignant TTs is required.

After treatment, children are monitored with physical examination, scrotal ultrasonography and tumoural markers [2, 32, 33].

The high resolution of ultrasonography imaging enables the detection of an increasing number of incidental impalpable testicular lesions often smaller than 5–10 mm. It is not possible to differentiate between benign or malignant small lesion, especially when clinical and tumoural markers are normal. No specific guidelines are currently available to manage small testicular lesions neither for adults nor for children. Based on the literature and our own experience, we propose that in children with a small (under 5 mm) incidental non-palpable testicular mass, follow-up US is sufficient. In case of a larger lesion (> 5 mm) or unclear ultrasound findings, a testis sparing surgery with an inguinal approach and extemporaneous frozen section analysis must be practised [16, 39, 44].

## Conclusion

TTs are rare in children. The benign tumours of the testis are more common in children and teratoma is the most frequent histological subtype. US has a sensitivity of almost 100% for the detection of a testicular mass. Elastography and CEUS are being studied and can be applied in future. MR is used as a supplemental imaging technique in exceptional cases where scrotal US findings are inconclusive or non-diagnostic and in the extension of a histologically confirmed malignant TT.

Testicular US, clinical and tumoural markers can help in the decision between orchiectomy or testis sparing surgery. Sometimes, intraoperative frozen section biopsy may be a determinant in the choice of the appropriate surgical procedure.

## Data Availability

Not applicable

## Abbreviations

AFP: Alpha-fetoprotein
CT: Computed tomography
MR: Magnetic resonance
TT: Testicular tumour
US: Ultrasonography
